# Increasing girls’ physical activity during an organised youth sport basketball program: a randomised controlled trial protocol

**DOI:** 10.1186/1471-2458-14-383

**Published:** 2014-04-21

**Authors:** Justin M Guagliano, Chris Lonsdale, Gregory S Kolt, Richard R Rosenkranz

**Affiliations:** 1 School of Science and Health, University of Western Sydney, Sydney, Australia; 2 Institute for Positive Psychology and Education, Australian Catholic University, Sydney, Australia; 3 Department of Human Nutrition, Kansas State University, Manhattan, Kansas, USA

**Keywords:** Moderate-to-vigorous physical activity, Inactivity, Youth sport, Organized sport, Training, Coach, Children, Adolescents, Coach education

## Abstract

**Background:**

Participation in organised youth sports (OYS) has been recommended as an opportunity to increase young peoples’ moderate-to-vigorous physical activity (MVPA) levels. Participants, however, spend a considerable proportion of time during OYS inactive. The purpose of this study, therefore, was to investigate whether coaches who attended coach education sessions (where education on increasing MVPA and decreasing inactivity during training was delivered) can increase players’ MVPA during training sessions over a 5-day basketball program compared to coaches who did not receive coach education sessions.

**Methods/design:**

A convenience sample of 80 female players and 8 coaches were recruited into the UWS School Holiday Basketball Program in Greater Western Sydney, Australia. A two-arm, parallel-group randomised controlled trial was employed to investigate whether coaches who attended 2 coach education sessions (compared with a no-treatment control) can increase their players’ MVPA during training sessions over a 5-day basketball program. Objectively measured physical activity, directly observed lesson context and leader behaviour, player motivation, players’ perceived autonomy support, and coaching information (regarding training session planning, estimations on player physical activity and lesson context during training, perceived ability to modify training sessions, perceived importance of physical activity during training, intention to increase physical activity/reduce inactivity, and likelihood of increasing physical activity/reducing inactivity) were assessed at baseline (day 1) and at follow-up (day 5). Linear mixed models will be used to analyse between arm differences in changes from baseline to follow-up on all outcomes.

**Discussion:**

The current trial protocol describes, to our knowledge, the first trial conducted in an OYS context to investigate the efficacy of an intervention, relative to a control, in increasing MVPA. This study’s findings will provide evidence to inform strategies targeting coaches to increase MVPA in OYS, which could have major public health implications, given the high proportion of children and adolescents who participate in OYS globally.

**Trial registration:**

This trial is registered with the Australian New Zealand Clinical Trials Registry, ACTRN12613001099718.

## Background

Globally, high proportions of children and adolescents participate in organised youth sports (OYS) [1-3]. In Australia, yearly prevalence data indicate that approximately 69% of children (67% of girls) participate in at least one OYS (including dance) outside of school hours [4]. An array of physical and psychosocial health and developmental benefits are associated with children and adolescents’ participation in OYS including, but not limited to, skill development, muscular strength and endurance, increased self-esteem, and positive peer relationships [5]. Given the high proportion of children who participate in OYS, coupled with myriad health and developmental benefits associated with sports participation, OYS has the potential to be a powerful health-promoting environment for children and adolescents.

One of the most pertinent attributes of OYS is its potential to contribute considerably to levels of moderate-to-vigorous physical activity (MVPA) in children [6,7]. Given that a sizeable proportion of children and adolescents do not meet the recommended 60 minutes of daily MVPA [8-11], participating in OYS could have a major impact on public health outcomes related to activity levels. This is particularly important for girls, as research shows that they are less physically active than boys [8,10], with the most pronounced declines in physical activity participation observed in adolescence [10].

Although OYS may provide an ideal opportunity for children and adolescents to accumulate substantial amounts of MVPA, studies have found that children and adolescents spend large proportions of time during OYS inactive or in light physical activity [6,12,13]. Furthermore, using a direct observation system [14], Guagliano, Rosenkranz, and Kolt [6] observed that coaches spent a considerable proportion of training time managing and instructing their players, time when children and adolescents would be relatively inactive. There is potential, then, for coaches to be able to influence their players’ physical activity levels; particularly during training where coaches are better able to dictate the intensity of physical activity, as compared to during a game. That said, to our knowledge, no study has used coaches to promote physical activity in OYS. One study, however, explored OYS coaches’ perceptions on this topic and it appears that coaches have the potential to be ideal candidates to promote physical activity in OYS [15]. Most coaches in the aforementioned study considered themselves role models for physical activity and felt it was part of their role as a coach to promote a fun, friendly, and supportive team environment that provided players with sport-specific development (physical and tactical skills) [15].

Recently, there has been a call to evaluate strategies for increasing MVPA in OYS [12]. The current trial protocol presents, as far as we are aware, the first randomised controlled trial (RCT) to be conducted in an OYS context aimed at determining the efficacy of coach education on MVPA. The primary aim of this two-armed RCT was to assess whether coaches who attended coach education sessions (where education on increasing MVPA and decreasing inactivity during training was delivered) could increase their players’ MVPA during training sessions over a 5-day basketball program compared to coaches who did not receive coach education sessions. The secondary aims were to: (1) assess whether players who were coached by coaches who have attended coach education sessions spent a lower percentage of time inactive during training sessions compared to players who are coached by coaches who did not attend coach education sessions; and (2) to investigate motivational effects on player physical activity. We also investigated changes in coaches’ awareness of player physical activity, how time was spent during training (lesson context), and leader behaviour.

Compared with a standard-care control coached as normal, we hypothesised that players who have been coached by coaches who have attended coach education sessions will: (1) spend a greater percentage of time in moderate-to-vigorous physically activity, (2) spend a lower percentage of time inactive, and (3) not exhibit lower motivation scores. Also, we hypothesised that coaches who have attended coach education sessions will have a greater awareness of their players’ physical activity.

## Methods/design

### Trial design

**Figure 1:**
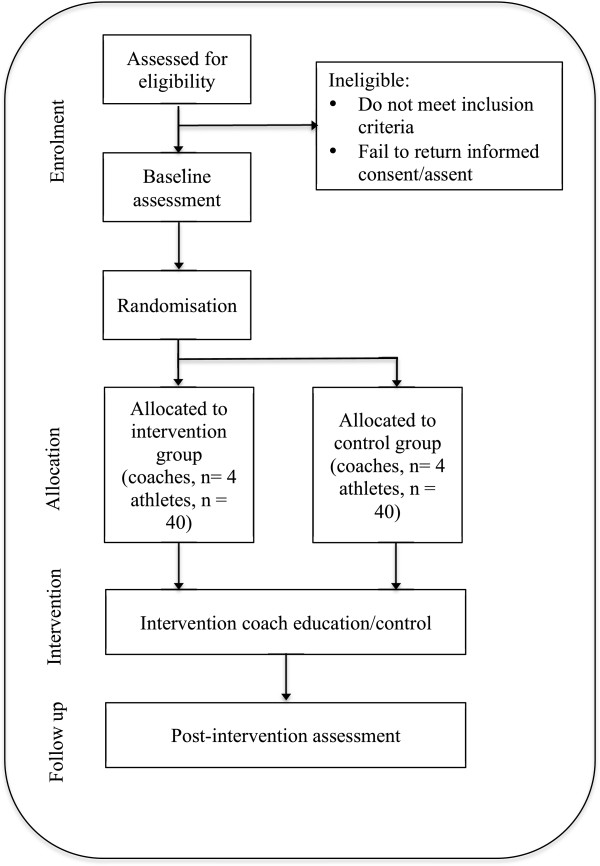
Flow diagram of study protocol.

This study is a two-armed, parallel-group RCT, using a 1:1 allocation ratio, designed to investigate whether coaches who attended coach education sessions (where education on increasing MVPA and decreasing inactivity during training was delivered) can increase their players’ MVPA during training sessions over a 5-day basketball program; compared to coaches who did not receive coach education sessions (Figure 1). Outcomes were assessed at baseline (day 1 of the basketball program) and follow up (day 5 of the program). The Human Research Ethics Committee of the University of Western Sydney (UWS) approved this study. This study adheres to the Consolidated Standards of Reporting Trials guidelines [16].

### Participants

We planned to recruit a convenience sample of 80 female players and 8 coaches into the UWS School Holiday Basketball Program. Players were recruited through the distribution of flyers to 5 OYS basketball clubs, 6 primary schools (private and Catholic), 3 community centres, 2 after-school programs, and social media (Yammer and E-update, which are private social networks for UWS staff, were used). Coaches were recruited via flyers to 2 OYS basketball clubs and the UWS (undergraduate students). To be considered eligible for this study as a player, participants needed to be female, aged 9–12 years, and intend to attend the program for its duration. For coaches to be eligible to coach in the program, basketball coaching credentials from the Australian Sports Commission’s National Coaching Accreditation Scheme (NCAS) [17] and previous experience coaching girls basketball teams were required. Coaches were also informed that participation might involve attending 2 coach education sessions; however, no information was divulged regarding what the coach education sessions entailed. Coaches also received payment for their time, at a rate of AUD$25/hour (intervention coaches were also paid to attend coach education sessions at the same rate).

### Sample size and power calculation

On the basis of an α of 0.05 and 80% power to detect a significant differential change in MVPA between groups, using an effect size of *d* = 0.6, a minimum sample size of 36 female players for each group was needed (*N* = 72). Our effect size is consistent with the findings of a recent systematic review and meta-analysis of interventions designed to increase children and adolescents’ MVPA in a similar setting (physical education), *d* = 0.62 [18]. To protect against player attrition and preserve adequate statistical power, the sample size was inflated by 10%, thus a total sample of 80 female players was sought.

### Blinding

Research assistants, blinded to study hypotheses and treatment allocation, conducted baseline assessments prior to randomisation. Players were also blinded to study hypotheses and treatment allocation. After baseline assessments and randomisation, 4 coaches were asked to attend coach education sessions (intervention) and 4 coaches served as controls; therefore, it was not possible to keep coaches blinded in this study. Lastly, a member of the research team who was blinded to participant (player and coach) allocation conducted all analyses.

### Randomisation

Coaches were randomly assigned to the site they were coaching by using simple randomisation; a computer-generated algorithm was used, ensuring an equal number of coaches at each site. Players, however, were not randomly allocated to a site; instead parental preference in site determined where the player would attend the basketball program, and this was predominantly based on location of the venue in relation to their residence.

Group randomisation for both players and coaches occurred following baseline assessments. Coaches were pair-matched using the average step counts their group of players accumulated during two training sessions during baseline assessments (i.e., the two coaches with the two highest group step count averages during the training sessions and the two coaches with the two lowest group step count averages during the training sessions were paired together). Coaches were pair-matched to ensure that similar coaches (in terms of the average group step counts accumulated by their players during the baseline training sessions) were randomised into each arm of the study. Given that increasing MVPA was our primary outcome and that pedometry has been shown to be an accurate indicator of MVPA [19], matching coaches via group step counts was appropriate. Using a computer-generated algorithm one coach from each pair was allocated into the intervention arm and the other into the control arm.

**Figure 2:**
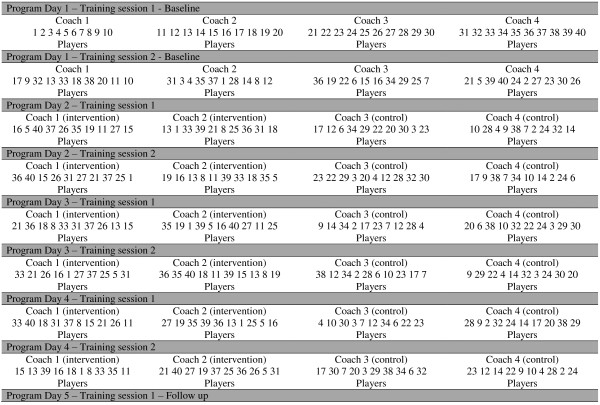
**Illustrates the randomisation procedure for each training session for one site.** Note: numbers represent player identification number.

Players at each site were randomly assigned using simple randomisation to either the intervention or control arm through a computer-generated algorithm, ensuring equal groups. To avoid clustering effects associated with having the same coach in each session throughout the program; players were randomised into different training groups and coaches within their allocated arm for each training session period for the duration of the program. Figure 2 illustrates the randomisation procedure for each training session for one site.

### Study procedure

The UWS School Holiday Basketball Program is a basketball program for girls that ran for 5 consecutive days, for 4 hours per day, over the school holiday period in September 2013 (Australian Spring). The basketball program ran simultaneously across 2 sports centres in Greater Western Sydney, Australia, with each site having 2 full-size basketball courts. At each site, we aimed to recruit 40 female players and 4 coaches and the data collection team comprised 1 supervisor and 4 research assistants.

Parent/guardians who wanted to register their daughter(s) in the basketball program, and coaches who wanted to coach in the basketball program, initiated contact with the primary researcher (JMG) expressing their interest. Initial contact was made via phone, text message, email, or face-to-face. JMG screened all interested participants for eligibility using a standardised script or email/message. Parents of players who were deemed eligible for inclusion were given a study information sheet, informed consent/assent form, an emergency contact form, and a parent questionnaire (described below) to complete. Coaches who were deemed eligible for inclusion were provided with a study information sheet (containing a basic description of primary study aim), informed consent form and a coach questionnaire (described below) to complete. When all forms were completed and returned, the participant (player or coach) was enrolled into the study.

The program was structured the same each day (see Table 1) and included 2 training sessions and 2 games. In each of the training sessions, coaches were instructed to focus on 2 skills; however, the coach planned their own training sessions to teach these skills. Each day, the first training session focused on dribbling and defending skills and the second training session focused on passing/catching and shooting skills. Coaches had half of a court to deliver their training session. During each training session, research assistants used the System for Observing Fitness Instruction Time (SOFIT) [14] to collect lesson context and coach behaviour data. During this time, players and coaches also wore sealed pedometers, and research assistants recorded their step counts at the conclusion of each training session. These data were summarised and entered onto a coach feedback form, where intervention coaches received a group average step count per minute and the percentage of time spent in each lesson context and coach behaviour according to SOFIT. This feedback was given to intervention coaches at the end of each day of the basketball program. A double round-robin tournament was created for the 2 games per day, which was played on a full court. During the designated breaks, players were free to do as they chose (e.g., talk amongst each other, eat, play basketball or other games of their choice).

**Table 1:** Daily program schedule

**Activity**	**Duration (in minutes)**
Training session 1	45
Break	15
Game 1	40
Break	15
Training session 2	45
Break	15
Game 2	40

Baseline assessments were collected on the first day and follow up assessments were collected on the fifth day of the basketball program (see Table 2 for a summary of the data collected). Following the first 2 days of the program, the 4 coaches allocated to the intervention arm of the study attended a coach education session.

**Table 2:** Summary of data collected

**Data collected**	**Data collection instrument**	**When data were collected**	**Data collection day**
Physical activity levels	Accelerometry	Duration of each program day	1-5
	Pedometry	Duration of each training session	1-5
	SOFIT	Duration of each training session	1-5
Lesson context	SOFIT	Duration of each training session	1-5
Coach behaviour	SOFIT	Duration of each training session	1-5
Player motivation	Situational Motivation Scale	Following training session 2	1,5
Players’ perceived autonomy support	Teacher as Social Context Questionnaire	Following training session 2	1,5
Players’ anthropometric data	Stadiometer, scale, tape measure	Baseline	1
Player demographic data	Parent questionnaire	Prior to study commencement	N/A
Coach demographic data	Coach questionnaire	Prior to study commencement	N/A
Coaching data	Coach questionnaire	Prior to study commencement/following training session 2	Before day 1, 3, 5
Process evaluation	Process evaluation	End of coach education session 2	2

Note. SOFIT = System for Observing Fitness Instruction time.

### Intervention

Although this study is the first intervention study in an OYS setting directed at increasing players’ MVPA, there have been several interventions conducted in a similar setting (physical education) with the same objective (see Lonsdale et al. [18] for a recent systematic review and meta-analysis). Several intervention studies included in Lonsdale et al.’s systematic review and meta-analysis incorporated similar intervention components as our intervention. For example, strategies to reduce management and instruction time [20,21] in an effort to reduce inactivity, create leader awareness [20], modified drills where physical activity was more inherent [22], and preparation/organisation [21,23]). The authors’ findings indicated that physical education based interventions can increase the proportion of time students spent in MVPA while participating in physical education lessons [18].

In the current study, coaches allocated into the intervention arm attended 2 coach education sessions. Each coach education session was approximately 2 hours in duration and took place in the afternoon following each of the first 2 days of the program. JMG conducted both coach education sessions. JMG is working towards a doctoral degree in the area of physical activity promotion in children and youth and has 4 years of experience coaching OYS teams. A research assistant was also present during both coach education sessions. The research assistant holds a doctoral degree in the area of physical activity promotion and also has previous experience (3 years) coaching OYS teams.

During the first coach education session (after day 1 of the program), approximately 20 minutes was spent providing coaches with information about MVPA (i.e., what it MVPA and how much should children accumulate daily based on national guidelines [24]). Further, JMG summarised findings of a previous study that examined girls’ MVPA in OYS (in terms of proportion of practice time and steps/min) [6]. Coaches were informed of a study by Scruggs, who found that 82–88 steps/min was approximately equivalent to spending 50% of the time physically active (in physical education) [25]. In a physical education setting, spending 50% time in MVPA has been recommended as a target [26]. Since no such recommendation exists in OYS, and a similar proportion of time spent in MVPA has been found in OYS [6] and physical education [27], we adopted Scruggs’ steps/min estimation as a guide for intervention coaches to gauge their athletes’ physical activity during the two training sessions. Coaches, however, were not explicitly instructed to aim for a specific proportion of time in MVPA or steps/min. Coaches were also shown how training time was typically spent during based on Guagliano et al.’s findings [6]. The aforementioned study [6] broke training sessions down into 6 mutually exclusive categories (management, knowledge delivery, fitness, skill practice, game play, and free play) based on the SOFIT [14] (described in detail below).

Coaches were then given roughly 15 minutes to reflect on their training sessions. Coaches were prompted to consider how active they thought their players were during training, how they spent their time during training, and potential modifications they could make to some of their drills to increase opportunities for MVPA.

Coaches were then presented with individualised feedback for each of their 2 training sessions. All information provided on the coaches’ feedback form was explained to the coaches by JMG (approximately 15 mins); which included: group average steps/minute, proportion of training time spent in each SOFIT lesson context (described in next section), and coach behaviour recorded as occurrences per session (described in next section).

The next 30 minutes were spent discussing potential strategies coaches could implement to increase opportunities for MVPA. More specifically, the importance of planning, conducting warm-ups and cool downs, dynamic stretching as opposed to static stretching, using small long-term groups, providing ample equipment, using circuits/grids as opposed to lines, and avoiding elimination games were discussed as potential strategies to increase opportunities for MVPA during training.

Coaches were then presented with a case study. The case study was a short video of a basketball training session. Coaches were asked to modify the drills in the video in order to increase MVPA. Once coaches had modified drills, each coach demonstrated their modified drill on a basketball court. Coaches had the remainder of the session to plan their training sessions for the next day (roughly 20 minutes).

The beginning of the second coach education session (after day 2 of the program) was devoted to reviewing the strategies to increase MVPA that were discussed in the first coach education session (about 10 minutes). Coaches then reflected on their training sessions for approximately 15 minutes (either alone or with one another). Coaches were prompted to reflect on the strategies they tried to incorporate into their training sessions (and their success in doing so), and similar to the first session, how active they thought their players were during training, and how they spent their time during training.

The next 30 minutes were spent discussing potential strategies coaches could implement to decrease inactivity during training. More specifically, JMG discussed potential strategies to decrease or modify management (e.g. drill transition or drink breaks) and instruction time to reduce inactive time. Self-monitoring (e.g., limit number of drills, limit number of times providing instruction, or, limit the time spent delivering instructions) and goal setting (e.g., setting proximal and distal goals for the basketball program) were also discussed as potential strategies that coaches could implement to decrease inactivity during their training sessions.

Similar to the first coach education session, coaches were presented with a video case study of a basketball training session and were asked to modify the drills in the video in order to increase MVPA or decrease inactivity. Coaches were also asked to modify some of their commonly used drills. Once coaches had modified drills, each coach demonstrated their modified drill to each other on a basketball court (approximately 30 minutes).The remaining time (about 35 minutes) was devoted planning their training sessions and completing a process evaluation questionnaire.

Intervention coaches continued to receive individualised feedback after each program day (i.e., on program days 3–5). Individualised feedback was furtively delivered to intervention coaches in an effort to avoid raising suspicion among control coaches.

Coaches allocated into the control arm of the study were asked to coach as usual. Control coaches had access to the same equipment (e.g., basketballs, pylons, coloured training jerseys) as intervention coaches, but were not privy to any information provided during the coach education sessions or individualised feedback.

### Outcome measures

#### Accelerometry

ActiGraph GT3X + accelerometers (ActiGraph; Pensacola, FL) were used to assess physical activity levels in this study. In a paediatric population, ActiGraph accelerometers have been shown to be valid and reliable devices for the measurement of physical activity levels [28,29]. Accelerometers were initialised once at the start of the week and set to record data at a sampling rate of 30 Hz, as well as step counts. Accelerometers were synchronized with an external clock and initialised to start recording 1 hour before the start of the first day of the basketball program and stop recording data 1 hour after the fifth day of the basketball program. Start and finish times of training sessions, games, and breaks were recorded. Players and coaches wore accelerometers. Female research assistants fitted players with an accelerometer. Accelerometers were placed over the right iliac crest and held in place using an adjustable elastic belt, prior to the start of each program day, and worn for the duration of the day. At the end of the fifth day, raw accelerometer counts were downloaded to a computer using ActiGraph software, integrated into 1-second epochs, and exported and saved to a Microsoft Excel file.

Evenson cut-points [30] have been recommended to estimate physical activity intensity in children and adolescents [29,31]. Freedson cut-points [32]; however, have been used by much of the existing literature that has examined physical activity in OYS [6,7,12]. Both cut-points were used in this study; Evenson cut-points were used as our primary outcome and Freedson cut-points were presented to facilitate comparisons with previous studies. Using Evenson cut-points [30], physical activity intensity was classified as the following (thresholds have been adjusted to account for 1-second epochs): inactive ≤1.67 counts per second; light physical activity ≥ 1.68 counts per second <38.25; moderate physical activity ≥38.26 counts per second <66.85; and vigorous physical activity ≥66.86 counts per second [30]. Using Freedson’s metabolic equivalent of task (MET) prediction equation [32] physical activity intensity was classified as the following: inactive ≤100 counts/min; light physical activity ≥ 1.5 METs <4; moderate physical activity ≥4 METs <7; and vigorous physical activity ≥7 METs [32]. To account for our 1-second epochs, age-specific counts per minute were divided by 60. Although there is still some debate regarding suitable MET-intensity thresholds for children and adolescents [29], the thresholds selected for this study have been previously used in a female paediatric population [6,33].

#### Pedometry

Two models of Yamax Digiwalker (Tokyo, Japan) pedometers were used in this study, the SW-200 and SW-700. Both the SW-200 and SW-700 models use the same pendulum mechanism to count steps [34]. Studies have found that the SW series of Yamax Digiwalkers is sensitive to increases in physical activity, has a high level of agreement with observed steps, and is a valid assessment of the volume of physical activity in children [35-37]. Players and coaches wore sealed pedometers over the right iliac crest, for the duration of both training sessions that occurred daily. Female research assistants assisted players with the placement of the pedometer. Research assistants recorded individual step counts following each training session and reset the pedometer. Pedometers were employed in this study to quickly provide intervention coaches with feedback on their players’ physical activity levels during that day’s training sessions.

#### Direct observation

SOFIT is a widely used direct observation system that uses momentary time sampling to generate data on players’ physical activity, lesson context, and leader behaviour [14]. Studies have shown that SOFIT has demonstrated acceptable reliability and validity in paediatric populations [14,38]. SOFIT can be easily implemented in an OYS setting, yet only one peer-reviewed study that we are aware of (conducted by our research team) has used the direct observation system in OYS [6].

Prior to session commencement, the observer implementing SOFIT, quasi-randomly and furtively selected 4 (plus an alternate) players to observe for the duration of the session [6,39]. Players were observed for 4 minutes at a time, on a rotational basis. Physical activity levels, lesson context, and leader behaviour were coded and recorded on paper every 20 seconds using a looped voice recording that prompted the observer to observe and record. At the end of each observe interval, lesson context was coded into only 1 of 6 mutually exclusive categories: management, knowledge delivery, fitness, skill practice, game play, and free play. Leader behaviour, however, is coded using a hierarchical format. Leader behaviour was coded into 1 of 4 categories and included (in hierarchal order) promotes physical activity (includes prompts of encouragement and praise) or discourages physical activity (includes prompts that are sarcastic and punitive in nature), demonstrates physical activity, and other. Promotes physical activity or discourages physical activity, therefore, is recorded if it occurs at any time during the 10-sec observe interval; whereas ‘other’ is only scored if the other categories are not observed during the 10-sec observe interval. JMG has been trained to use the observation technique and has collected SOFIT data for other peer-reviewed work [6]. JMG trained all research assistants to use SOFIT using recommended guidelines [40]. Research assistants’ SOFIT coding accuracy was assessed against a pre-coded ‘gold standard’ video developed by McKenzie [40]. Coding accuracy was assessed using percent agreement, where a minimum of 80% agreement between scores was set as the minimum acceptable level of agreement [40].

#### Questionnaires

At baseline (day 1) and follow up (day 5), players were asked to complete a questionnaire assessing their perceptions of their coach’s autonomy-supportive behaviour by completing 4 items from the Teacher as Social Context Questionnaire [41,42]. Players responded to questions on a 7-point Likert scale (1 = not true at all, 7 = very true). Scores on the TASC were averaged and ranged from 1 to 7, higher scores were indicative of greater perceived coach autonomy-supportive behaviour.

Players also completed the 14-item Situational Motivation Scale which assesses constructs of intrinsic motivation, identified regulation, external regulation, and amotivation [43]. Players responded to questions on a 7-point Likert scale (1 = not true at all, 7 = very true). Based on players’ average scores from the four subscales of the SIMS, a self-determination index (SDI) was created (SDI = 2*intrinsic motivation + identified motivation – external regulation – 2*amotivation, e.g., Lonsdale et al. [44]). Scores on the SIMS can range from −18 to 18, where higher scores were indicative of greater self-determined motivation towards participation in a situation (i.e., basketball practice) [44,45]. Both the Teacher as Social Context Questionnaire and the Situational Motivation Scale have received empirical support for reliability and validity [41,46,47].

A demographic questionnaire was distributed to parents and coaches for descriptive data purposes. The questionnaire that was distributed to parents collected data on parents’ level of education, relationship status, and household income. The questionnaire also collected data on their daughter’s age, country of birth, cultural background, and OYS information (number of OYS played, level, number and minutes of training sessions per week, and number and minutes of games per week). This questionnaire was only distributed to parents once, prior to the commencement of the study.

The questionnaire distributed to coaches collected data on: age, sex, height, weight, country of birth, cultural background, highest education qualification, relationship status, OYS information (number of OYS played, level, number and minutes of training sessions per week, and number and minutes of games per week), physical activity information (number and time spent in vigorous, moderate, and light physical activity) and leisure-time information (time spent sleeping, sitting, standing, watching television, and using a computer). These data were only collected from coaches once, prior to commencement of the study. Coaches responded to questions on a 5-point Likert scale (1 = not at all, 5 = to a great extent) about coaching, regarding training session planning, estimations on player physical activity during training, estimations on percentage of time spent in each SOFIT lesson context (described above), perceived ability to modify training sessions, perceived importance of physical activity during training, intention to increase physical activity/reduce inactivity, and likelihood of increasing physical activity/reducing inactivity. These data were collected prior to the start of the study, after intervention end (day 3), and at follow up (day 5).

#### Anthropometric measures

Prior to measurement, players were asked to remove shoes and any heavy clothing. Standing height was measured to the nearest 0.1 cm using a portable stadiometer (PE87 portable stadiometer; Mentone Educational, Victoria, Australia). Weight was measured using a digital scale (EF 538 HealthStream digital scale; Aussie Fitness, Queensland, Australia) to the nearest 0.1 kg. Using the Centers for Disease Control and Prevention growth charts, body mass index (BMI) was calculated and converted into age- and sex-specific percentiles [48]. Waist circumference measurements were taken on the right side of the body by finding the midpoint between the lowest rib and the iliac crest [49]. A non-elastic tape measure (Myotape; Mentone Educational, Victoria, Australia) was wrapped snugly around the waist and measurement was taken at the end of exhalation to the nearest 0.1 cm. All measurements were conducted in duplicate and an average was recorded. A third measurement was taken if the first two measures differ by more than 0.5 cm or 0.5 kg and the average was recorded. Female research assistants collected all waist circumference measurements.

#### Process evaluation

A process evaluation was undertaken following the UWS School Holiday Basketball Program. The process evaluation assessed, using questions on a 5-point Likert scale (1 = not at all, 5 = to a great extent) and open-ended questions, the program’s feasibility and acceptability of the program amongst coaches.

### Statistical analysis

All variables will be checked for normality using the Shapiro-Wilk test. Independent samples *t-*tests or Mann–Whitney *U-*tests will then be conducted, as appropriate, to examine: (1) baseline differences between groups and (2) baseline differences between players who completed the study and those lost to follow-up. If variables significantly differ between groups, they will be appropriately adjusted in the main analyses.

Linear mixed models will be used to analyse the differential change between groups on all outcomes from baseline to follow-up, using baseline data as the covariate. Linear mixed models will be used because these models are robust enough to withstand the biases from missing data, and provide good control of Type I and Type II errors [50]. All analyses will be conducted using SPSS 21.0 (Chicago, IL, USA). The level of significance will be set at *p* < 0.05.

## Discussion

A number of studies have recently been published examining OYS clubs as a setting to promote health [51-53]. These studies illustrate the wide range of health-promoting capabilities OYS can provide and the importance of OYS clubs, yet, only one study has acknowledged that OYS clubs could play a role in promoting physical activity [53]. Studies have shown that children and adolescents can accumulate considerable amounts of MVPA during OYS; however, the majority of time spent during OYS is either inactive or in a light physical activity intensity [6,7,12]. Thus, there is clearly an opportunity to optimise MVPA levels and reduce inactivity and light physical activity during OYS. If the intervention is successful, this study’s findings will support the use of coach education sessions to increase MVPA in OYS, which can have major public health implications given the high proportion of children and adolescents who participate in OYS globally [1-4].

This study is not without its limitations. First, this study only investigated the short-term efficacy of coach education on player physical activity as the basketball program took place over five consecutive days. Further, by conducting this study as an OYS basketball program (rather than using players’ usual competition teams and coaches) may have limited our generalisability; however, it has increased our internal validity by allowing us to test the efficacy of the intervention in a more tightly controlled environment. The decision to conduct this study in the form of an OYS basketball program (rather than using players’ usual competition teams and coaches) was made to allow us to individually randomise players to different training groups and coaches within their allocated arm each training session period. If this study were conducted in players’ usual competition, sampling would have had to take place on three levels (player, coach, OYS club), each additional level, an additional source of sampling error causing power to drop. By forming an OYS basketball program and individually randomising players to different training groups and coaches, we avoided a clustering effect and thus a cluster randomised controlled trial study design that would require a much larger sample. Studies conducted in a similar setting have observed high intra-class correlations for MVPA (indicating a large clustering effect) [45,54]. Our sample size, then, would have been inflated considerably to reach sufficient power and account for the clustering; which would not have been feasible. Despite this limitation, the present study employed a rigorous study design and used a high-resolution (1-second epochs) objective measurement to assess physical activity. Additionally, this study will provide insight on girls’ motivation, lesson context and leader behaviour (through SOFIT).

The current trial protocol presents, as far as we are aware, the first intervention to be conducted in an OYS context designed to investigate the efficacy of coach education sessions (relative to a no-treatment control) on increasing players’ MVPA and reducing inactivity during training. If the intervention is successful, this study’s findings will support the use of coach education sessions to increase MVPA in OYS; thus, this study’s protocol can be used as a starting point to inform future interventions and strategies to increase MVPA and reduce inactivity during OYS.

## Abbreviations

MVPA: Moderate-to-vigorous physical activity; OYS: Organised youth sport; RCT: Randomised controlled trial; SDI: Self-determination index; SOFIT: System for Observing Fitness Instruction Time; UWS: University of Western Sydney.

## Competing interests

The authors declare that they have no competing interests.

## Authors’ contributions

All authors conceived the project and contributed to the study design. JMG managed data collection, performed sample size calculations, and drafted the manuscript. CL, GSK, and RRR reviewed and edited the manuscript. All authors approved the final manuscript.

## Pre-publication history

The pre-publication history for this paper can be accessed here:

http://www.biomedcentral.com/1471-2458/14/383/prepub
